# DNA Methylation Profiling at Single-Base Resolution Reveals Gestational Folic Acid Supplementation Influences the Epigenome of Mouse Offspring Cerebellum

**DOI:** 10.3389/fnins.2016.00168

**Published:** 2016-05-03

**Authors:** Subit Barua, Salomon Kuizon, W. Ted Brown, Mohammed A. Junaid

**Affiliations:** ^1^Departments of Developmental Biochemistry, New York State Institute for Basic Research in Developmental Disabilities, Staten Island, NY, USA; ^2^Human Genetics, New York State Institute for Basic Research in Developmental Disabilities, Staten Island, NY, USA

**Keywords:** folic acid, DNA methylation, gestational development, brain development, cerebellum, psychiatric disease

## Abstract

It is becoming increasingly more evident that lifestyle, environmental factors, and maternal nutrition during gestation can influence the epigenome of the developing fetus and thus modulate the physiological outcome. Variations in the intake of maternal nutrients affecting one-carbon metabolism may influence brain development and exert long-term effects on the health of the progeny. In this study, we investigated whether supplementation with high maternal folic acid during gestation alters DNA methylation and gene expression in the cerebellum of mouse offspring. We used reduced representation bisulfite sequencing to analyze the DNA methylation profile at the single-base resolution level. The genome-wide DNA methylation analysis revealed that supplementation with higher maternal folic acid resulted in distinct methylation patterns (*P* < 0.05) of CpG and non-CpG sites in the cerebellum of offspring. Such variations of methylation and gene expression in the cerebellum of offspring were highly sex-specific, including several genes of the neuronal pathways. These findings demonstrate that alterations in the level of maternal folic acid during gestation can influence methylation and gene expression in the cerebellum of offspring. Such changes in the offspring epigenome may alter neurodevelopment and influence the functional outcome of neurologic and psychiatric diseases.

## Introduction

DNA reprogramming is essential for early embryonic development; around the time of implantation, *de novo* methylation is initiated in embryonic cells and is required for complete embryonic development (Li et al., 1992; Lei et al., 1996; Okano et al., 1999; Dean et al., 2001). In mammals, cytosine methylation is highly prevalent at CpG islands that modulate the chromatin structure and binding of transcriptional factors at promoter regions (Jaenisch and Bird, 2003). Thus, alterations in DNA methylation and epigenetic modification that occur during a specific window of gestational development can dysregulate gene expression and are associated with many diseases (Oberlander et al., 2008; Stein et al., 2009; Suter et al., 2010; Gore et al., 2011; Li et al., 2013; Perkins et al., 2013; West et al., 2013; Vanhees et al., 2014). Studies in humans and animal models have shown that variations in intake of the maternal nutrients involved in one-carbon metabolism, including folic acid (FA), during pregnancy can induce persistent changes in the offspring's epigenome and modulate various physiological outcomes (Cooney et al., 2002; Bean et al., 2011; Boeke et al., 2012; Greenop et al., 2014; O'Neill et al., 2014; Barua and Junaid, 2015). Our earlier studies in a mouse model had shown that exposure to high FA supplementation during gestation causes widespread changes in the methylation and gene expression in the cerebral hemisphere of the offspring (Barua et al., 2014b). Moreover, such exposure during gestation and the post-weaning period resulted in moderate changes in behavior (Barua et al., 2014a).

Over the past decades, several studies have shown that the cerebellum (CB) plays a significant role in coordination to motor functions and is involved in various cognitive processes, including perception, attention, and emotional behavior (Leiner et al., 1991; Martin et al., 2003; Schmahmann and Caplan, 2006). Recent studies with post-mortem brain samples have shown widespread aberrant methylation and gene expression in the CB of psychotic patients (Chen et al., 2014). Studies with a mouse model and in human post-mortem CB of individuals with autism have shown altered patterns of DNA methylation (Shpyleva et al., 2014), and indeed, cerebellar abnormalities have been reported in more than 95% of post-mortem examinations of individuals with autism (Marzban et al., 2014). To investigate whether higher supplementation with a methyl diet during gestation impacts the cerebellar development of offspring, in this study, we tested the hypothesis that higher folic acid supplementation during gestation can alter the methylation and gene expression in the CB of offspring.

## Materials and methods

### Animals and experimental design

All animal experiments were performed in accordance with protocols reviewed and approved by the Institute for Basic Research Institutional Animal Care and Use Committee in conformity with the NIH Guide for Care and Use of Laboratory Animals (NIH publication No. 86-23, revised 1985). One week prior to mating and throughout gestation, adult 8–10 week-old C57BL/6 J female mice were fed a custom AIN-93G amino acid–based diet (Research Diet, Inc., North Brunswick, NJ), having either low maternal folic acid (LMFA), at 0.4 mg/kg (*n* = 12), or high maternal folic acid (HMFA), at 4 mg/kg (*n* = 12). These levels of FA supplementation were chosen in this study, as women with a prior history of complicated and neural tube defect (NTD)–affected pregnancy are recommended to take 10-fold higher FA (4 mg/day) in comparison to other pregnant women (400–800 μg/day). FA at the 0.4 mg/kg diet level is necessary for a normal healthy litter, whereas FA at the 4 mg/kg diet level is 10 times higher.

### Tissue collection and processing

At post-natal day one (P1), pups from different dams were sacrificed, and CB tissues were dissected. The numbers of tissues collected from the LMFA group were: male pups, *n* = 15, and female pups *n* = 15. The numbers of tissues collected from the HMFA were: male pups *n* = 15, and female pups, *n* = 15. Tissues were immediately stored at −70°C until further use. From these, tissues were distributed for subsequent DNA/RNA and protein analysis.

### DNA extraction

CB tissues were extracted from P1 pups and pooled (*n* = 3/gender/group, each from an independent dam). DNA was extracted with the Epicenter MasterPure DNA purification kit (Epicenter Biotechnologies, Madison, WI, USA) by following the manufacturer's instructions, and concentration was measured by using a NanoDrop ND-1000 (Thermo Scientific, Wilmington, DE, USA).

### Library construction, sequence alignments, and data analysis

Library construction, sequence alignments, and data analysis were performed by following the detailed protocol previously described (Barua et al., 2014b). Libraries were prepared from 200 to 500 ng of genomic DNA after sequential digestion with 60 units of TaqI and 30 units of MspI (New England Biolabs, Ipswich, MA, USA), and sequencing was performed on an Illumina HiSeq genome analyzer. Sequence reads from bisulfite-treated EpiQuest libraries were identified using standard Illumina base-calling software and then analyzed using a Zymo Research proprietary analysis pipeline, which is written in Python. Bismark (http://www.bioinformatics.babraham.ac.uk/projects/bismark/) was the alignment software in the analysis pipeline. Index files were constructed by *bismark_genome_preparation* command using the entire reference genome. –*non_directional* and all the other default parameters were applied for running Bismark. Filled-in nucleotides were trimmed off when doing methylation calling. The number of reads reporting a C was divided by the total number of reads reporting a C or T was used to estimate the methylation level of sample cytosine. For each CpG site, Fisher's exact test was performed, which covered at least five reads. Moreover, for each CpG promoter, gene body and CpG island annotations were added. The total numbers of reads that were taken into account for each CpG site are given in Tables **2**, **3**, and Tables S1–S4 (column total CpG). All the procedures above were carried out in the Zymo Epigentic Core Services (Zymo Research, Irvine, CA). Sequence data has been deposited at the Sequence Read Archive (accession number SRX1608467) in the National Center for Biotechnology Information (NCBI).

### Quantitative real-time polymerase chain reaction (qPCR) analysis

Total RNA was extracted (*n* = 12, segregated by gender) by lysing the cells with Trizol reagent (Life Technologies, Inc., Carlsbad, CA) and was further purified by Qiagen RNeasy kit (Qiagen, Valencia, CA), according to the manufacturer's protocol as described earlier (Barua et al., 2014c). For each sample, on-column DNase digestion was performed to remove any DNA contamination. Quantitative RT-PCR was performed with the One-Step iScript kit (BioRad, Hercules, CA) or the Two-step kit (Affymetrix, Santa Clara, CA) by following the manufacturer's instructions. *Hprt1* was used as the endogenous control, and the relative expression was calculated using the Pfaffl method. For each gene, the mRNA expression was measured from *n* = 3/gender/group, each from an independent dam. Each reaction was run in triplicates, and only the expression of those genes that exhibited the same directional changes in independent pools from at least two independent animals was considered significant. The statistical difference between samples was determined by Student's *t*-test by using Prism Software (GraphPad, San Diego, CA); values are presented as means ± standard deviation. Primers used for qRT-PCR are listed in Table S5.

### Western blot analyses

Total cell lysates (*n* = 16) from the CB of male (M) and female (F) pups were prepared from both the HMFA and the LMFA groups. For each of these two groups, four male CBs (each male pup from an independent dam) and four female CBs (each female pup from an independent dam), for a total eight pups were analyzed. Western blot analyses were done as previously described (Barua et al., 2014c). Targeted proteins were detected by incubating with primary antibodies overnight at 4°C (dilutions for anti-GAD1 1:500 and anti-PARK2 1:200) followed by the secondary antibody coupled with horseradish peroxidase. Densitometric evaluation of the bands was calculated by using ImageJ software (NIH) and was normalized to the densities of β-actin staining as housekeeping control. The average calculated ratios of target protein to β-actin are presented.

## Results

### Global DNA methylation patterns of the offspring's CB from HMFA

The statistics of the mapping of the methylation profiles of pups CB's of pups from mothers supplemented with LMFA and HMFA are given in Table 1. The ratio of mapped reads to total reads ranged from 40 to 46.34% in male pups and from 56.87 to 61.33% in female pups from the LMFA and HMFA groups, respectively, with an average depth of CpG coverage (12x–13x) in males and (10x–16x) in females. The bisulfate conversion rates were approximately 98% for all the samples. Analysis of the global methylation profile revealed that 19% of the CpG sites were differentially methylated in pups from the HMFA group in comparison to that of the LMFA group in both male and female pups (*n* = 40.376 for male and *n* = 44.974 for female). Moreover, the majority of differentially methylated regions (DMRs) were in the intergenic or introns, whereas 19–20% were in exons, and 8–9% were in the promoter regions in the CpG-island sequence (Figures S1A,B). Similar to the CpG sites, the distribution of DMRs in the non-CpG sites revealed that the majority of DMRs were in the intergenic or introns, whereas 9–10% in exons, and 15–23% in the promoter regions (Figure S2). The distribution of methylation ratios and the Pearson's correlation coefficient for the corresponding CpG and non-CpG sites of male and female pups are shown in Figures S3, S4. The hexbin plot (Figures S5–S7) shows the distribution of overlapped CpG and non-CpG sites (*P* < 0.05) in male and female pups as a result of HMFA. The result of global DNA methylation profiling indicated that HMFA altered the methylation pattern of the epigenome of the offspring's CB.

**Table 1:** **Descriptive statistics of the mapping of methylation profile of pups' cerebellums from mothers supplemented with FA at 0.4 (LMFA) and 4 mg/kg (HMFA)**.

**Cerebellum**	**Total read**	**Mapped read**	**Mapping ratio (%)**	**Unique CpG**	**CpG Cov. (X)**	**BS conv. rate (%)**
CB-04 Male	29,885,144	13,848,250	46.34	4,430,390	12	98.09
CB-4 Male	29,016,284	11,607,234	40.00	3,348,964	13	98.70
CB-04 Female	37,174,068	21,140,555	56.87	4,643,308	16	98.59
CB-4 Female	17,432,488	10,690,857	61.33	3,784,838	10	98.78

### Maternal FA alters DNA methylation status of several genes in the CpG and non-CpG sites in offspring's CB

DNA methylation analysis at the single base level revealed that HMFA resulted in significant alterations in the methylation level of several genes. The alterations of methylation level were found in both CpG and non-CpG (CHG, CHH) sites throughout the entire genome in both male and female pups from HMFA group (Figures S1, S2). Such alterations were evident both in promoter and gene body regions and resulted in either hyper-methylation or hypo-methylation (*P* ≤ 0.05) in pups supplemented with HMFA. Multiple testing corrections revealed that HMFA resulted in hyper-methylation (Table 2) of several genes that modulate neuronal pathways in male pups (*Atp1a1, Kcnq4, Bre, Scnn1a, Celsr3, Kcnk10*), whereas the methylation of a gene of the neurexin gene family (*Nrxn2*) was hyper-methylated in female pups. Further analysis of methylation data revealed significant hypo-methylation (Table 3) of several genes in both male and female pups from the HMFA group. Several genes related to intellectual disability (*Dcaf17, Myst4, Park2, Rbfox1*) were found to be hypo-methylated in male pups, and genes (*Pfn1, Cntnap1, Drp2*) related to normal function of the nervous system were hypo-methylated in female pups from the HMFA group. Transcriptional factors in male pups (*Gtf2i, Nr3c2*) and in female pups (*Foxl2, Rfx1*) were hyper-methylated, whereas transcriptional factor *Rn45s* was hypo-methylated in female pups. Of note, the methylation level of sidekick cell adhesion molecule 1 (*Sdk1*) was hyper-methylated in both male and female pups from the HMFA group.

**Table 2:** **List of hypermethylated CpG/CHH/CHG sites in the gene body/promoter/other chromosomal region of genes from high maternal folic acid diet that were significantly altered after multiple testing corrections**.

**Chromosome**	**Start**	**End**	**Gene**	**Total CpG LMFA**	**Total CpG HMFA**	**Methylation difference**	***P-*value**	**Adj *P-*value**
**MALE**								
**CpG**								
Chr3	101396425	101396426	*Atp1a1*	48	55	0.69	4.73043E-11	8.61543E-06
Chr4^a^	120370725	120370726	*Kcnq4*	38	18	0.73	5.23985E-08	0.000602429
Chr5	32310326	32310327	*Bre*	8	16	1.00	1.35967E-06	0.00766814
Chr5^a^	122872838	122872839	*Anapc7*	20	11	0.90	9.21198E-07	0.005834628
Chr6^a^	99642691	99642692	*Gpr27*	46	10	0.80	2.59437E-06	0.012370994
Chr6	125280040	125280041	*Scnn1a*	21	33	0.68	8.58529E-08	0.000857291
Chr7	29537833	29537834	*Sars2*	17	17	0.76	5.1294E-06	0.020249774
Chr8	79656982	79656983	*Nr3c2*	30	24	0.68	2.79663E-07	0.002218414
Chr9	108751023	108751024	*Celsr3*	32	20	0.70	4.85931E-07	0.003592574
Chr11^a^	4962328	4962329	*Gas2l1*	14	18	0.89	5.09083E-07	0.003724971
Chr12	3739974	3739975	*Dtnb*	15	20	0.77	1.10085E-05	0.034438481
Chr12^a^	52930473	52930474	*Hectd1*	31	11	0.91	7.47565E-09	0.000114327
Chr12	83903228	83903229	*Rgs6*	28	41	0.71	2.68793E-11	8.35061E-06
Chr12	99800383	99800384	*Kcnk10*	14	32	0.69	9.50913E-06	0.03190158
ChrX	110293664	110293665	*Chm*	6	24	1.00	1.68414E-06	0.008878596
**CHH**								
Chr3	37380544	37380545	*Spata5*	10	16	1.00	1.88262E-07	0.018712081
Chr3	37379114	37379115	*Spata5*	39	54	0.52	4.92021E-09	0.000825025
Chr5^a^	122872790	122872791	*Anapc7*	57	38	0.71	2.29993E-11	5.49912E-05
Chr5	129289390	129289391	*Rimbp2*	22	42	0.62	3.47908E-07	0.031282409
Chr5	142522151	142522152	*Sdk1*	41	52	0.49	2.95614E-07	0.027788529
Chr8	34944721	34944722	*Rbpms*	33	40	0.59	1.33163E-07	0.013543758
Chr11	89851744	89851745	*Pctp*	46	39	0.52	6.98215E-08	0.007876096
Chr17	81118969	81118970	*Map4k3*	20	36	0.72	6.36563E-08	0.007313615
						0.00		
**CHG**						0.00		
Chr4	108146626	108146627	*Zcchc11*	112	115	0.34	5.53559E-08	0.006775161
Chr5	134721807	134721808	*Gtf2i*	57	80	0.49	9.5847E-11	7.42648E-05
Chr5	73446765	73446766	*Fryl*	64	49	0.44	1.56966E-08	0.002561524
Chr8	34914184	34914185	*Rbpms*	34	62	0.52	3.10146E-08	0.004794897
**FEMALE**								
**CpG**								
Chr2	18865431	18865432	*Pip4k2a*	14	8	1.00	3.12725E-06	0.017610194
Chr4	150525065	150525066	*Camta1*	17	14	0.81	6.84434E-06	0.032886803
Chr7	133845071	133845072	*Coro1a*	89	14	0.70	5.12126E-07	0.003733256
Chr8	86608595	86608596	*Rfx1*	45	12	0.70	9.53995E-06	0.042482671
Chr8^*^	86608595	86608596	Mir709^*^	45	12	0.70	9.53995E-06	0.042482671
Chr10	79397201	79397202	*Arid3a*	29	14	0.93	1.53102E-09	1.95146E-05
Chr10	60250203	60250204	*Unc5b*	25	18	0.70	5.43229E-06	0.027663076
Chr11	102139305	102139306	*Asb16*	34	19	0.68	1.40228E-06	0.009072618
Chr14	52609319	52609320	*Arhgef40*	42	8	0.90	9.21996E-07	0.006230346
Chr14^*^	52609319	52609320	Gm16617^*^	42	8	0.90	9.21996E-07	0.006230346
Chr16	10979333	10979334	*Litaf*	38	10	0.95	1.00906E-08	0.00011098
Chr17^a^	39985399	39985400	*Rn45s*	20	11	0.90	9.21198E-07	0.006230346
Chr18	75697791	75697792	*Ctif*	58	11	0.74	5.0559E-06	0.025932481
Chr19^a^	6428759	6428760	*Nrxn2*	52	10	0.72	5.74263E-06	0.028897735
ChrX^a^	137133982	137133983	*Tsc22d3*	39	8	0.95	1.43104E-07	0.001162634
ChrX^a^^*^	53984977	53984978	Fhl1^*^	46	14	0.83	1.84349E-08	0.000189568
ChrX^a^^*^	160346929	160346930	Ap1s2^*^	52	10	0.72	5.74263E-06	0.028897735
**CHH**								
Chr19^a^	46072916	46072917	*9130011E15Rik*	61	6	1.00	1.00205E-08	0.00773454
**CHG**								
Chr9	57880963	57880964	*Ccdc33*	53	6	1.00	2.21939E-08	0.012821301
Chr9^a^	98856591	98856592	*Foxl2*	102	11	0.64	8.54473E-09	0.006910752
Chr9^a^^*^	98856591	98856592	Foxl2os^*^	102	11	0.64	8.54473E-09	0.006910752
Chr19^a^	37625255	37625256	*Exoc6*	66	8	0.97	2.98577E-09	0.003018518
**OTHER CHROMOSOMAL REGIONS**								
**MALE**								
**CpG**								
Chr2	117020483	117020484		19	23	0.72	5.75229E-06	0.022192691
Chr4	55291226	55291227		24	17	0.69	1.55743E-05	0.044508695
Chr6	122591925	122591926		17	23	0.71	1.1076E-06	0.006597209
Chr10	92819632	92819633		16	31	0.81	3.98075E-09	7.03909E-05
Chr12	110057962	110057963		16	31	0.75	8.42303E-07	0.00545744
Chr14	87019484	87019485		14	26	0.78	1.29013E-06	0.007324618
Chr17	80418706	80418707		31	23	0.75	5.72815E-09	9.24604E-05
Chr17	80418649	80418650		31	23	0.74	2.14082E-09	4.08331E-05
Chr17	80418656	80418657		31	23	0.74	2.14082E-09	4.08331E-05
Chr17	80418710	80418711		31	22	0.73	5.02628E-09	8.44787E-05
Chr17	80418721	80418722		28	23	0.70	1.02512E-07	0.000977631
Chr17	49319822	49319823		18	47	0.68	8.1497E-07	0.005341511
Chr18	8736817	8736818		32	9	0.81	1.4286E-05	0.04188451
Chr18	8736774	8736775		31	9	0.81	1.83039E-05	0.048931908
Chr18	60275673	60275674		14	22	0.71	3.93805E-06	0.017229446
**CHH**								
Chr3	37105839	37105840		28	60	0.50	4.59752E-07	0.039616449
Chr8	32211528	32211529		45	70	0.57	5.4005E-11	5.49912E-05
Chr8	29104849	29104850		30	44	0.55	1.11592E-07	0.011734506
Chr8	27826273	27826274		36	48	0.51	5.73672E-07	0.0474555
Chr17	85245770	85245771		36	62	0.68	9.22107E-11	5.49912E-05
**CHG**								
Chr3	18872260	18872261		69	40	0.45	6.79721E-10	0.000153587
Chr9	4249915	4249916		41	86	0.53	5.27759E-10	0.000129188
**FEMALE**								
**CpG**								
Chr3	15582599	15582600		64	28	0.73	3.54618E-11	1.16245E-05
Chr7	142676163	142676164		50	6	0.92	6.46782E-06	0.03164917
Chr7	140397403	140397404		46	20	0.67	2.57852E-07	0.001995863
Chr11	116362692	116362693		50	6	0.96	8.62376E-07	0.005883499
ChrX	139577966	139577967		8	18	0.76	0.000397497	0.506460372
ChrX	118333137	118333138		44	13	0.68	8.19276E-06	0.037584248
**CHH**								
Chr8	114228185	114228186		43	6	1.00	7.15112E-08	0.047312275
**CHG**								
Chr18	83201037	83201038		70	12	0.67	1.38883E-08	0.009360419

a Methylation exclusively in the CpG island;

* Methylation in the promoter regions; HMFA, high maternal folic acid; LMFA, low maternal folic acid. CHH, CHG where H can be A, T or G.

**Table 3:** **List of hypomethylated CpG/CHH/CHG sites in the gene body/promoter/other chromosomal region of genes from high maternal folic acid diet that were significantly altered after multiple testing corrections**.

**Chromosome**	**Start**	**End**	**Gene**	**Total CpG**	**Total CpG**	**Methylation**	***P-*value**	**Adj *P-*value**
				**LMFA**	**HMFA**	**difference**		
**MALE**								
**CpG**								
Chr2	70926781	70926782	*Dcaf17*	12	8	−1.00	7.9384E-06	0.028133219
Chr3	51477712	51477713	*Mgst2*	16	15	−0.73	1.6121E-05	0.045534315
Chr5	111715613	111715614	*Ttc28*	46	19	−0.79	1.95557E-09	3.9055E-05
Chr10	79393579	79393580	*Arid3a*	18	12	−0.83	2.1967E-06	0.010935514
Chr11	69574236	69574237	*Amac1*	42	15	−0.80	2.10416E-08	0.000279056
Chr11	89384648	89384649	*Ankfn1*	16	19	−0.74	9.65017E-06	0.032243587
Chr12	109176310	109176311	*Bcl11b*	10	31	−0.77	1.73473E-05	0.04752513
Chr13	24228093	24228094	*Lrrc16a*	13	16	−0.86	3.07969E-06	0.014240168
Chr17	11729485	11729486	*Park2*	10	10	−1.00	1.08251E-05	0.034194413
**CHH**								
Chr5	150366342	150366343	*Wdr95*	27	32	−0.85	1.21698E-12	7.55036E-06
Chr6	119463258	119463259	*Wnt5b*	16	43	−0.69	1.56071E-08	0.002104991
Chr8	119603496	119603497	*Pkd1l2*	25	56	−0.60	7.02256E-09	0.001146559
Chr9	107166117	107166118	*Mapkapk3*	24	39	−0.65	2.17476E-07	0.020757812
Chr16	6736334	6736335	*Rbfox1*	26	36	−0.71	4.2963E-09	0.000740418
						0.00		
**CHG**						0.00		
Chr4	43744421	43744422	*5430416O09Rik*	38	79	−0.66	1.89728E-11	4.03799E-05
Chr9	103033308	103033309	*Rab6b*	23	37	−0.65	9.21745E-09	0.00159268
Chr9	104021165	104021166	*Acad11*	38	72	−0.58	2.74934E-11	4.03799E-05
Chr11	118130307	118130308	*Usp36*	38	51	−0.55	2.20911E-10	7.42648E-05
Chr16	15797072	15797073	*Prkdc*	8	26	−1.00	5.50776E-08	0.006775161
**FEMALE**								
**CpG**								
Chr1^a^	39592922	39592923	*D1Bwg0212e*	46	12	−0.72	7.29815E-06	0.034293206
Chr2	54854875	54854876	*Galnt13*	40	10	−0.85	7.79574E-07	0.005370225
Chr3	152572664	152572665	*St6galnac5*	46	10	−0.76	1.32097E-06	0.008651764
Chr5	150883968	150883969	*Rxfp2*	52	6	−0.92	5.18834E-06	0.026484159
Chr5	4009104	4009105	*Akap9*	47	8	−0.87	2.4664E-06	0.014583234
Chr7	13564506	13564507	*Zfp446*	17	6	−1.00	9.90619E-06	0.043500018
Chr7	142277612	142277613	*Dock1*	49	15	−0.71	2.05041E-08	0.000206849
Chr8^a^	73985459	73985460	*Abhd8*	66	11	−0.76	1.60125E-06	0.01005433
Chr9	45614148	45614149	*Cep164*	41	15	−0.80	8.14852E-10	1.23014E-05
Chr9	65575164	65575165	*Zfp609*	35	8	−0.75	4.59283E-06	0.024030758
Chr11	100924353	100924354	*Atp6v0a1*	40	6	−0.95	2.98928E-06	0.016967907
Chr11	63783989	63783990	*Cox10*	24	12	−0.92	7.27024E-08	0.00063948
Chr11	118386794	118386795	*Rbfox3*	33	10	−0.82	4.17663E-06	0.02232177
Chr11	70466348	70466349	*Pfn1*	71	18	−0.75	3.48721E-09	4.17015E-05
Chr11	101045277	101045278	*Cntnap1*	84	16	−0.71	4.67003E-08	0.00043033
Chr13	115629726	115629727	*Itga2*	52	14	−0.70	1.73075E-06	0.010740731
Chr15	73386877	73386878	*Dennd3*	42	6	−0.95	2.28171E-06	0.013758747
Chr15	76684078	76684079	*Zfp251*	43	7	−0.71	9.91146E-06	0.043500018
Chr17	26145026	26145027	*Rab11fip3*	35	6	−1.00	2.22401E-07	0.00174687
Chr17	34201190	34201191	*Col11a2*	69	10	−0.71	4.42664E-06	0.023265511
Chr18	64349640	64349641	*1700091E21Rik*	60	8	−0.72	6.76961E-06	0.032675219
ChrX	130978215	130978216	*Drp2*	42	6	−0.95	2.28171E-06	0.013758747
**CHG**								
Chr17^a^	39981459	39981460	*Rn45s*	215	1801	−0.32	3.15966E-11	0.000127773
**OTHER CHROMOSOMAL REGIONS**								
**MALE**								
**CpG**								
Chr4	114927822	114927823		19	16	−1.00	2.4631E-10	1.6251E-05
Chr4	70356713	70356714		11	8	−1.00	1.32307E-05	0.039611439
Chr4	33145068	33145069		22	23	−0.74	1.15388E-07	0.001082193
Chr6	22702718	22702719		20	38	−0.79	1.68255E-09	3.40026E-05
Chr8	23561782	23561783		10	9	−1.00	1.08251E-05	0.034194413
Chr8	125951782	125951783		6	15	−1.00	1.84284E-05	0.049033126
Chr8	15641954	15641955		33	10	−0.87	1.72635E-07	0.001510603
Chr9	49862607	49862608		10	10	−1.00	1.08251E-05	0.034194413
Chr10	12602192	12602193		17	8	−1.00	9.24578E-07	0.005834628
Chr10	123989391	123989392		17	35	−0.83	4.59988E-09	7.96787E-05
Chr12	44576005	44576006		19	14	−0.80	3.041E-06	0.014104497
Chr12	68850704	68850705		31	14	−0.76	1.20506E-06	0.007029716
Chr14	13972178	13972179		44	9	−0.84	4.38892E-07	0.003296639
Chr15	31799762	31799763		14	16	−0.81	4.79293E-06	0.019439463
Chr16	55473911	55473912		24	11	−0.78	1.52675E-05	0.043927676
Chr17	84101994	84101995		10	10	−1.00	1.08251E-05	0.034194413
Chr17	40509054	40509055		26	26	−0.73	1.66363E-07	0.001486366
**CHH**								
Chr4	62532417	62532418		30	18	−0.80	3.6826E-08	0.004662758
Chr16	39097237	39097238		47	34	−0.70	4.56498E-11	5.49912E-05
Chr18	53985910	53985911		27	30	−0.63	6.04117E-08	0.007071799
**CHG**								
Chr4	44486603	44486604		24	72	−0.53	1.53357E-07	0.017325988
Chr8	97707113	97707114		31	43	−0.52	4.46831E-08	0.006250153
Chr8	91367262	91367263		39	56	−0.46	5.61123E-09	0.001030159
Chr10	93725790	93725791		84	53	−0.46	1.1065E-10	7.42648E-05
Chr11	101785985	101785986		26	44	−0.54	4.99742E-08	0.006672518
**FEMALE**								
**CpG**								
Chr1	72001078	72001079		32	8	−0.75	7.29474E-06	0.034293206
Chr2	178977613	178977614		52	10	−0.88	7.44799E-08	0.000649745
Chr3	69055711	69055712		66	8	−0.85	2.90337E-06	0.016568628
Chr5	48149488	48149489		54	10	−0.80	1.01668E-08	0.00011098
Chr7	76060069	76060070		43	10	−0.77	9.4751E-06	0.042282364
Chr8	4203056	4203057		66	6	−0.91	5.91402E-06	0.029620167
Chr8	66000281	66000282		61	8	−0.72	6.1866E-06	0.030412915
Chr16	22049549	22049550		26	14	−0.71	1.1809E-06	0.007855174
Chr18	83282753	83282754		95	9	−0.83	9.90991E-08	0.000823993
Chr18	67611225	67611226		62	10	−0.74	9.90604E-06	0.043500018
Chr19	45266101	45266102		30	18	−0.80	3.6826E-08	0.00034839

a Methylation exclusively in the CpG island; HMFA, high maternal folic acid; LMFA, low maternal folic acid. CHH, CHG where H can be A, T or G.

Further analysis of all *P* ≤ 0.05 differential methylation sites without correction for multiple testing identified several genes that were hyper-methylated or hypo-methylated in male and female pups, in both the CpG and non-CpG sites (Tables S1–S4). The methylation level of genes associated with autism spectrum disorder (*Plxna4, Arid1b, Kdm4c, Runx1, Accn1, Aff2, Chd9, Cntnap2, Grip1, Grin2b*, and *Mid1*); imprinted genes (*Peg12, Tsix*); transcriptional factors (*Ebf2, Lmx1b, Runx3, Sox13*, and *Mef2a*) that modulate neurogenesis; and genes related to neurodevelopment (*Grik4, Ntrk2, Sgk1, Cacna1a, Gabrg3, Erbb3*, and *Gfra1*) were found to be altered in CB of both male and female pups from the HMFA group. Our findings suggest that maternal diet during gestation, specifically HMFA, can modulate the methylation profile of several genes, including those involved in neural development in the gene bodies and the promoter, CpG, and non-CpG sites in the CB of pups' brains.

### Maternal FA alters expression of several differentially methylated genes in offspring's CB

**Figure 1:**
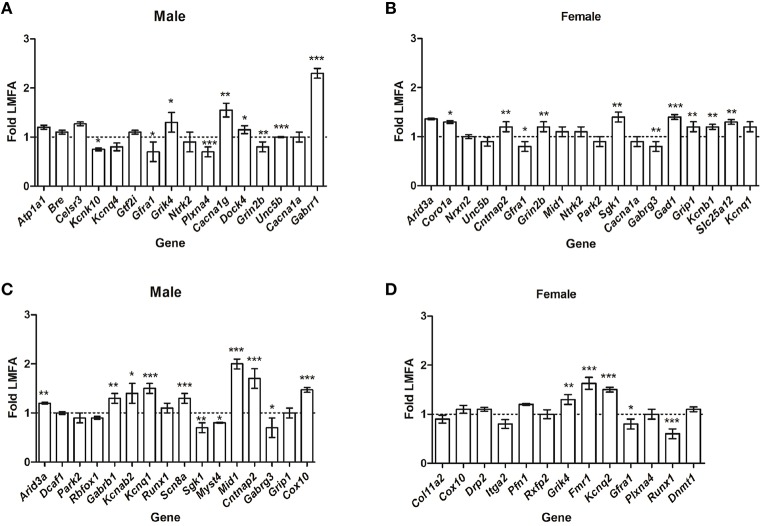
**Relative expression of the genes that exhibited hyper-methylation (A,B) and hypo-methylation (C,D)**. The results were normalized to *Hprt* transcript expression and were expressed as relative values in comparison with corresponding transcripts from low maternal folic acid (LMFA). Results represent mean ± standard deviation (SD); asterisks denote statistically significant change (^*^P < 0.05, ^**^P < 0.01, ^***^P < 0.001).

To extend our findings, we then analyzed whether HMFA induced changes in the overall methylation profile in offspring's CB correlates with the alterations in gene expression. Quantitative RT-PCR analysis of several genes that showed differential methylation (*P* ≤ 0.05) exhibited variations in expression in pups from the HMFA group. Genes in male pups from the HMFA group that exhibited significant hyper-methylation after multiple testing corrections in CpG sites (*Atp1a1, Bre, Celsr3, Kcnq4*) and *Gtf2i* in CHG sites did not exhibit any changes in expression level compared to pups from the LMFA group (Figure 1A). In contrast, expression of *Kcnk10*, a gene that encodes protein from the potassium channel family and is hyper-methylated in CpG sites, was significantly down-regulated in male pups from the HMFA group (Figure 1A). In female pups from the HMFA group, genes that exhibited hyper-methylation in CpG sites after multiple corrections (*Arid3a, Nrxn2, Unc5b*) exhibited no significant change in expression, whereas the expression of *Coro1a* was up-regulated significantly (Figure 1B). Similarly, analysis of expression of genes that were hypo-methylated at CpG sites by HMFA revealed significant up-regulation in expression of *Arid3a* in male pups whereas the expression of several other genes in male (*Dcaf17, Park2, Rbfox1*) and in female (*Col11a2, Cox10, Drp2, Itga2, Pfn1*, and *Rxfp2*) pups did not exhibit any significant changes (Figures 1C,D).

To further reveal the impact of maternal FA, we next assessed whether HMFA-induced changes of methylation (*P* ≤ 0.05, without corrections) alter the expression of genes related to neuronal pathways (Figures 1A–D). Our results showed significant down-regulation in the expression of genes (*Gfra1, Plxna4, and Grin2b*), up-regulation in the expression of genes (*Grik4, Cacna1g, Dock4, Unc5b*, and *Gabrr1*), and no changes in the expression level of genes (*Ntrk2, Cacna1a*) in male pups that exhibited hyper-methylation at CpG or non-CpG sites (Figure 1A). In female pups from the HMFA group, several genes that were hyper-methylated at CpG or non-CpG sites also exhibited changes in expression level (Figure 1B).

Although the expression of some genes (*Gfra1, Gabrg3*) was down-regulated and of other genes (*Cntnap2, Grin2b, Sgk1, Gad1, Grip1, Kcnb1*, and *Slc25a12*) was up-regulated, the expression of some other genes (*Mid1, Ntrk2, Park2, Cacna1a, Park2, and Kcnq1*) was unaltered. Similarly, expression analysis of genes that exhibited hypo-methylation (*P* ≤ 0.05, without corrections) in CpG and non-CpG sites revealed down-regulation in the expression of genes in male (*Sgk1, Myst4, Gabrg3*) and in female (*Gfra1, Runx1*) pups (Figures 1C,D). HMFA resulted in up-regulation in the expression of some genes in male (*Gabrb1, Kcnab2, Kcnq1, Scn8a, Mid1, Cntnap2, Cox10*) and in female (*Grik4, Fmr1, Kcnq2*) pups, whereas the expression of other genes in male (*Runx1, Grip1*) and in female (*Plxna4, Dnmt1*) pups remained unaltered (Figures 1C,D).

### Maternal FA down-regulates expression of gad1p in offspring's CB

**Figure 2:**
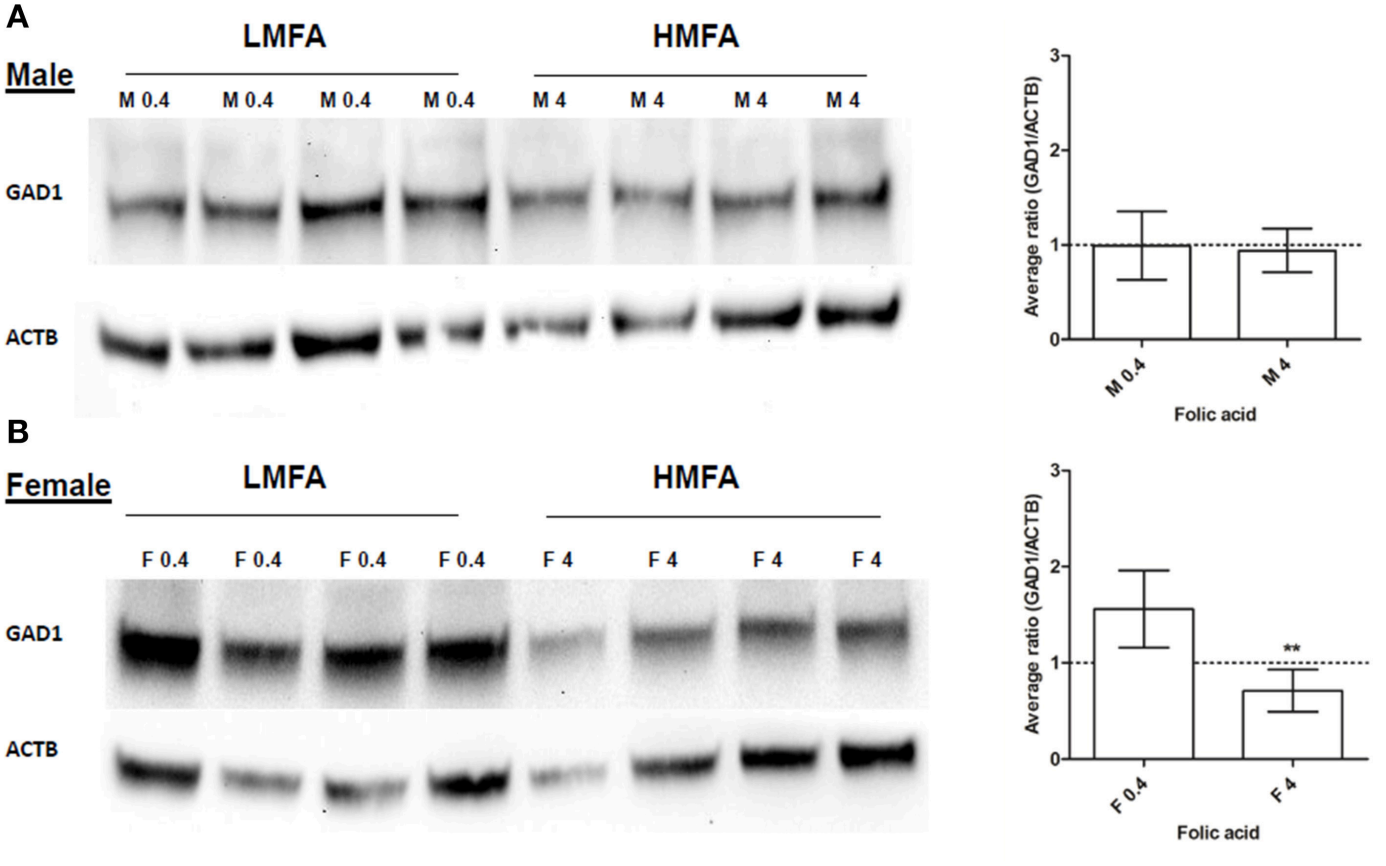
**Western blot analysis showing the expression of Gad1p in the cerebellum of (A) male (M) and (B) female (F) pups from LMFA (0.4 mg/kg diet) and HMFA (4 mg/kg diet) groups**. The left panel shows one representative blot, and the right panel shows the mean densitometric evaluation. The error bar ± SD represents the inter-variability among independent samples (*n* = 4). Asterisks denote statistically significant change (**P < 0.05) by unpaired *T*-test.

**Figure 3:**
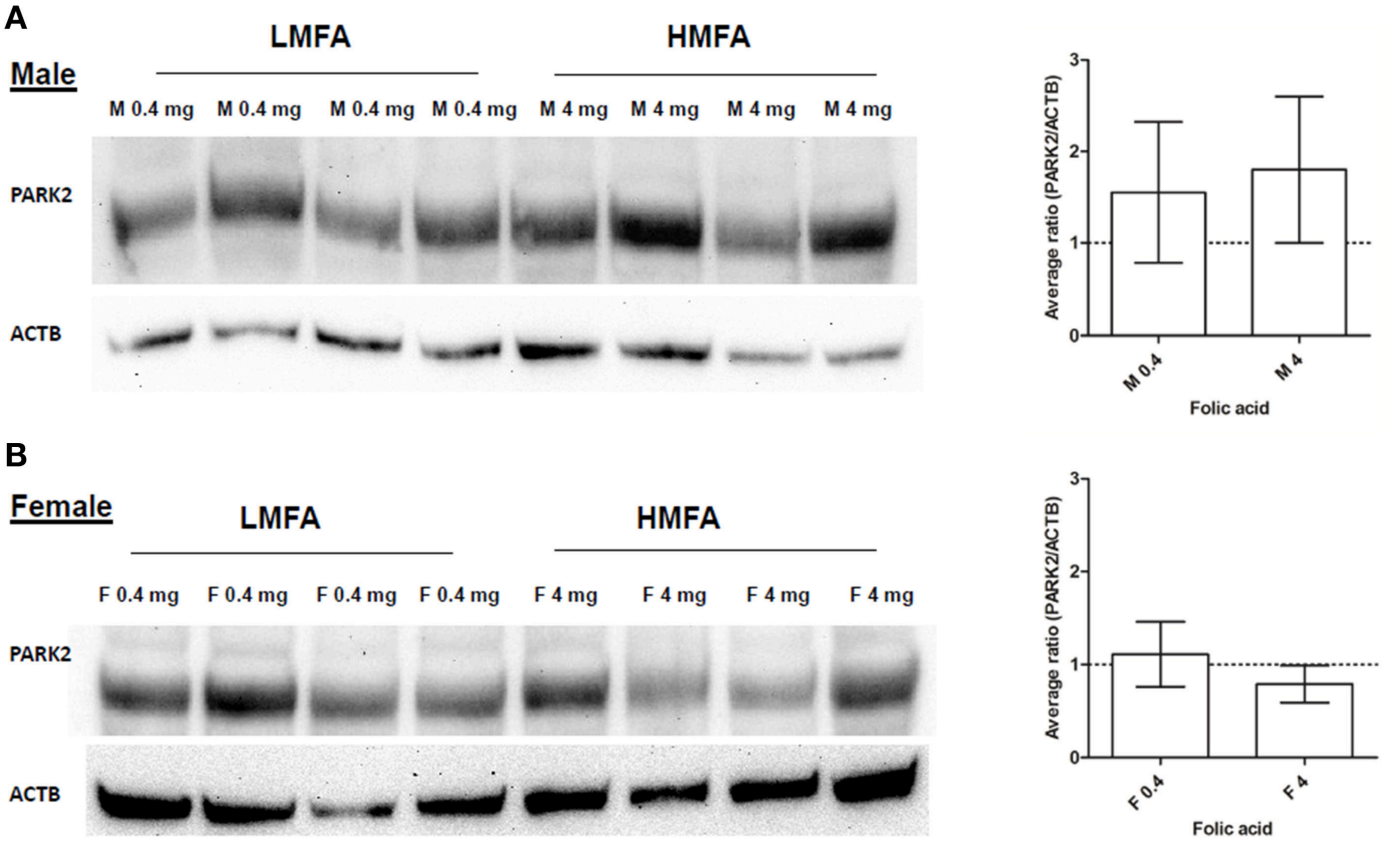
**Western blot analysis showing the expression of Park2p in the CB of (A) male and (B) female pups from LMFA (0.4 mg/kg diet) and HMFA (4 mg/kg diet)**. The left panel represents one representative blot, and the right panel shows the mean densitometric evaluation. The error bar ± SD represents the inter-variability among independent samples (*n* = 4).

Next, we analyzed whether the expression of two genes (*Gad1*, hyper-methylated at CpG in female pups and *Park2*, hyper- and hypo-methylated at both CpG and non-CpG sites in male and female pups) that play a role in neuronal pathways including autism are altered at the protein level. Western blot analysis and densitometric quantification of the protein levels revealed that the expression of Gad1p (Figure 2A) in male pups remains unaltered; however, the expression of Gad1p in female pups from the HMFA group was significantly reduced (Figure 2B). In contrast, the expression of Park2p remained unchanged in both male and female pups from the HMFA group (Figures 3A,B). These results show that HMFA during gestation can modulate the expression of genes in offspring's CB and that such impact can be gender-specific.

### Maternal FA modulates sex-specific alterations in expression of genes in the offspring's CB

**Figure 4:**
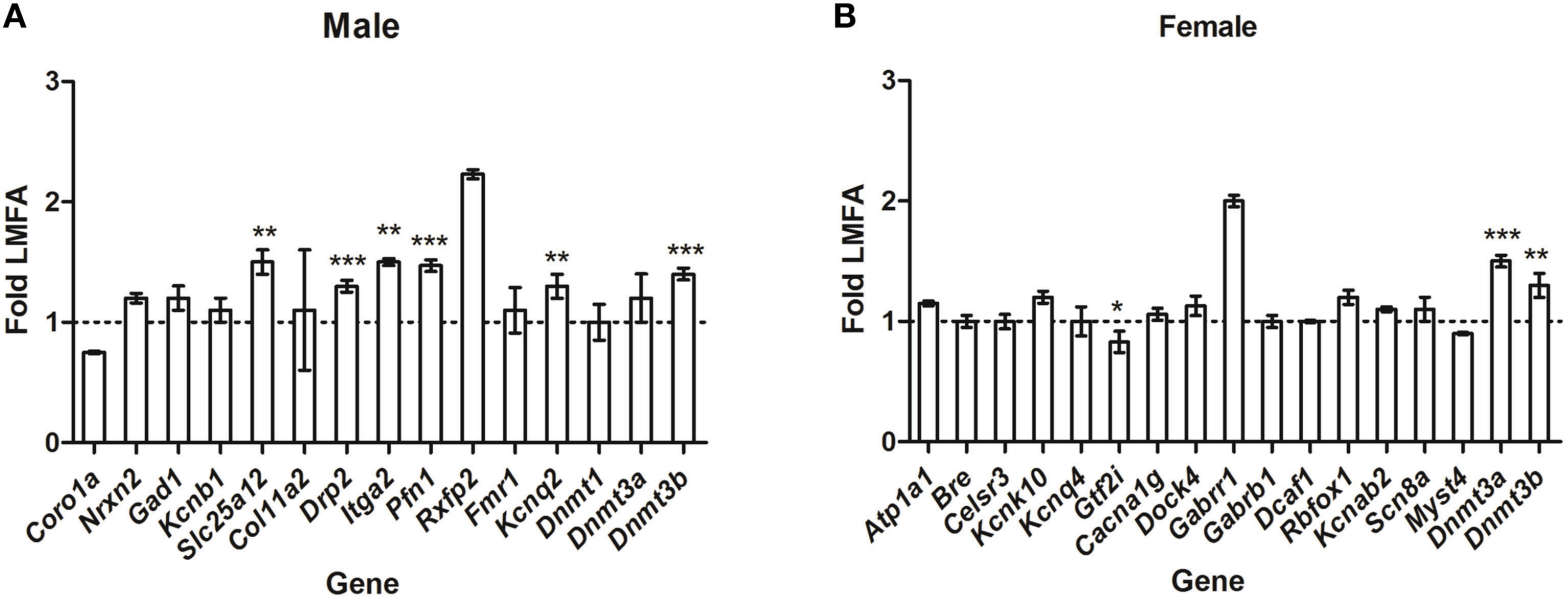
**Quantitative real time reverse transcription-polymerase chain reaction (qRT-PCR) showing relative expression of the transcripts of genes in (A) male pups and (B) female pups from the HMFA group that exhibited no alterations in the methylation profile in promoter and gene body in the cerebellum compared with the LMFA**. The results were normalized to Hprt transcript expression and were expressed as relative values in comparison with corresponding transcripts from LMFA. Results represent mean ± standard deviation (SD); asterisks denote statistically significant change (^*^P < 0.05, ^**^P < 0.01, ^***^P < 0.001).

To gain insight into whether HMFA-induced changes in methylation profiles modulate sex-specific alterations in the expression of genes, we analyzed the expression of several genes that exhibited alterations in methylation in the opposite sex. In male pups, the expression of the genes *Coro1a, Gad1, Kcnb1*, and *Fmr1* (which exhibited changes in methylation and gene expression in female pups) was not altered (Figure 4A); in contrast, the expression of *Drp2, Itga2*, and *Pfn1* was altered in male pups from the HMFA group, although it exhibited no alterations in methylation profile in comparison to the LMFA group. However, the expression of the genes *Nrxn2, Col11a2, Dnmt1*, and *Rxfp2* was unaltered; while *Slc25a12*, and *Kcnq2* was altered in pups of both genders from the HMFA group. Similarly, in female pups, the expression of genes (Figure 4B) *Kcnk10, Cacna1g, Dock4, Gabrr1, Gabrb1, Kcnab2, Scn8a*, and *Myst4* (which exhibited changes in methylation and gene expression in male pups) was not altered, in contrast to the expression of *Gtf2i* being altered in female pups from the HMFA group, although it exhibited no alterations in methylation profile in comparison to the LFMA group. However, expression of the genes *Atp1a1, Bre, Celsr3, Kcnq4, Dcaf1*, and *Rbfox1* was unaltered in pups of both genders from the HMFA group.

## Discussion

DNA methylation in the early embryo has long been associated with the maintenance of genomic integrity, with its crucial role in modulating gene expression and genomic imprinting (Farthing et al., 2008; Messerschmidt et al., 2014). Epigenetic alterations of effectors in the developing brains of mammals can impair normal development and result in neurodevelopmental disorders (Schanen, 2006; LaSalle and Yasui, 2009; LaSalle, 2011). In this study, we performed genome-wide methylation analysis to test the hypothesis that exposure to HMFA during gestation induces epigenetic changes in the mouse offspring's CB and contributes to alterations in gene expression. We found distinct alterations in the methylation profile in The CB of offspring from mothers that received HMFA in comparison to LMFA. Such changes were widespread throughout the genome and were evident in both promoter and intragenic regions. These data establish that in addition to its role in preventing NTD's, FA during gestation can induce epigenetic changes in the brain.

As a pteroylmonoglutamate, FA cannot breach the blood brain barrier, with only methylfolate appearing in the cerebrospinal fluid (CSF). After FA is consumed, metabolically it is chemically reduced to tetrahydrofolate by the enzyme dihydrofolate reductase (DHFR) in the liver, where it collects a formyl group, which is reduced to methyl to form 5-methyltetrahydrofolic acid (Barua et al., 2014c). However, excess supplementation with FA may overwhelm this process and possibly saturate the DHFR activity. Perhaps the benefit of high doses of FA may be limited by saturation of DHFR, as studies have shown that DHFR activity in human liver is extremely slow and variable (Bailey and Ayling, 2009). We speculate that mechanistically one possibility is that excess FA in blood may compete to bind with the folate receptor and inhibit methyl-tetrahydrofoalte transport, resulting in changes in brain homeostasis (Smith et al., 2008). Alternatively, elevated methylfolate via maternal metabolism may concentrate against a gradient in the CSF by the choroid plexus and thus interfere with the regulatory functions of the brain. Because the developing brain is potentially vulnerable to maternal nutrient availability (Antonow-Schlorke et al., 2011), such exposure to HMFA may impact the brain function of offspring with alterations in methylation and gene expression, as is evident from the data of our study.

To date, much progress has been made to characterize the dynamic changes of DNA methylation in CpG sites and its role in functional outcome. However, the research to elucidate the precise mechanism by which methylation in non-CpG sites affects gene expression and its role in functional outcomes is still in its infancy. Studies have shown that *Dnmts* have sequence specificity and can influence the methylation in non-CpG sites (Ramsahoye et al., 2000; Gowher and Jeltsch, 2001; Mund et al., 2004). Similar to our previous findings (Barua et al., 2014b), the data generated from this study suggest that alterations in FA intake during gestation can modulate the methylation profile of both CpG and non-CpG sites in the brains of offspring. Our results revealed that HMFA increases the expression levels of *Dnmt3b* in male pups and of *Dnmt3a, Dnmt3b* in female pups (Figure 4). Such evidence supports the idea that HMFA may increase the activity of *Dnmts* which can modulate neuronal differentiation (Luo et al., 2013); perhaps neuronal cells have a higher variation in DNA methylation than non-neuronal cells (Zhang et al., 2010; Melka et al., 2014). This study further establishes the theory of variations in methylation of neuronal cells, with distinct methylation patterns of CpG and non-CpG sites throughout the genome, including promoters and intergenic and intragenic regions in the CB of pups from the HMFA group in comparison to the LMFA group. This finding is in agreement with our previous reports and suggests that HMFA during gestation can induce unbiased distribution of methylation in the brains of offspring. Such differences in the methylation pattern in the intragenic region may have different correlations to the expression of transcriptional levels, as is evident from our study. Indeed, studies in mammalian cells and human brains correlated changes in methylation of intragenic regions with both up-regulation and down-regulation of transcripts (Ball et al., 2009; Rauch et al., 2009; Maunakea et al., 2010).

Our data also highlighted that several genes that play a role in neuronal pathways exhibited alterations in expression in the CB of offspring. For example, pups from HMFA exhibited significant changes in the expression of several voltage-dependent ion channels (*Cacna1g, Scna8a, Kcnk10, Kcnab2, Kcnq1, Kcnq2*), which may result in modulation in neuronal synaptic transmission and excitability (Vacher et al., 2008).

Similarly, several genes (*Grik4, Gabrr1, Gabrg3, Gabrb1*, and *Gad1*) involved in GABAergic (gamma-aminobutyric acid) or glutamatergic synaptic transmissions exhibited alterations in expression in pups from HMFA. Studies have reported that GABAergic interneurons play a central role in the modulation of excitatory output in the brain and have been documented with a wide variety of psychiatric diseases, including mood disorders, schizophrenia and autism (Fatemi and Folsom, 2014). Biochemical studies have revealed that the expressions of GAD65/67 are decreased in the CB of subjects with developmental disorders such as autism and schizophrenia (Bullock et al., 2008; Blatt and Fatemi, 2011). In the current study, we found that HMFA decreased the expression of Gad1p in female pups, where the expression in the male pups remains unaltered.

Such alterations in the expression of Gad1p may induce alterations in learning and social behavior, as studies with Gad67-deficient mice have been reported to exhibit behavioral abnormalities and reduced sociability (Sandhu et al., 2014; Zhang et al., 2014). Indeed, our previous studies showed that gestational and post-weaning HMFA resulted in changes in behavior, with an increase in ultrasonic vocalization as neonates (Barua et al., 2014a). Such findings indicate that maternal nutritional status, specifically variations in the methyl diet, can induce changes in DNA methylation and thus modulate the expression of genes involved in neuronal pathways and may impact the behavioral outcomes.

Off note, neuronal cells are found to exhibit more inter-individual variations compare to non-neuronal cells (Iwamoto et al., 2011). Moreover, studies with post-mortem brain samples from patient with major depression have shown significant cell specific epigenetic variation in between brain regions (Guintivano et al., 2013). One of the limitations of our current study is that we used the whole cerebellum from pups for methylation and expression analysis; thus some of the alterations of DNA methylation profile may results due to cellular heterogeneity.

Previous studies have reported sexual dimorphism and asymmetry in animal and human cerebellum (Ramirez and Jimenez, 2002; Fan et al., 2010). It is also reported that perinatal exposure to chemical and physical perturbations impact differential neurodevelopment and behavioral effects in males and females (Nguon et al., 2005); other studies with mouse placenta have shown that maternal diet impacts gene expression differentially in males and females (Mao et al., 2010; Gabory et al., 2012). Our data also shows a significant bias between genders in methylation and expression of genes in the offspring CB as a result of HMFA. This finding is congruent with our previous report that HMFA induces gender-specific methylation changes in the brains of offspring (Barua et al., 2014b). We speculate that such differential methylation could results from alterations in the uterine environment because of excess FA, and biased sensitivity to nutritional perturbations resulted from gender specific distribution of specific receptors and methylation of imprinted genes.

## Conclusions

Given the role of maternal FA in the methylation pathway, it remains an open questions how such epigenetic modifications impact the brain and behavior of offspring. Our findings support the idea that epigenetic variations may have distinct sex biased functional consequences to certain neuropsychiatric disorders. It further highlights the relevance of studying both sexes in both experimental model and clinical studies to study the epigenetic impact of maternal diet.

In summary, a key finding of this study is that FA during gestation, aside from its role in preventing NTDs, can induce alterations in the methylation of several genes in both CpG and non-CpG regions in the offspring's CB, and such alterations in methylation are gene- and sex- specific. Such changes in DNA methylation during gestation can induce alterations in gene regulatory structures, and given the role the CB in regulation of higher order functions, including motor function and cognition (Schmahmann, 2004; Tavano et al., 2007), such changes may modulate the functional outcome of neurologic and psychiatric diseases.

## Author contributions

WB and MJ conceived the experiments; SB, WB, and MJ designed the experiments; SB, SK, and MJ performed the experiments; SB and MJ analyzed the data; SB, SK, WB, and MJ contributed reagents/materials/analysis tools; SB wrote the paper, and WB and MJ critically revised the manuscript.

### Conflict of interest statement

The authors declare that the research was conducted in the absence of any commercial or financial relationships that could be construed as a potential conflict of interest. The reviewer SK and handling Editor declared their shared affiliation, and the handling Editor states that the process nevertheless met the standards of a fair and objective review.

## Supplementary material

Figure S1 **Distribution of differentially methylated sites in CpG island sequences. (A)** Male; low maternal folic acid (LMFA) vs. high maternal folic acid (HMFA). **(B)** Female LMFA vs. HMFA.

Figure S2 **Distribution of differentially methylated sites in non-CpG (CHG/CHH) sites of cerebellum. (A)** Male low maternal folic acid (LMFA) vs. high maternal folic acid (HMFA). **(B)** Female LMFA vs. HMFA.

Figure S3 **Scatter plot representing the distribution of the methylation ratio for corresponding sites (A) CpG, (B) CHG, and (C) CHH of LMFA vs. HMFA of male pups**. Pearson's correlation coefficient is denoted in the center of each scatter plot.

Figure S4 **Scatter plot representing the distribution of the methylation ratio for corresponding sites (A) CpG, (B) CHG, and (C) CHH of LMFA vs. HMFA of female pups**. Pearson's correlation coefficient is denoted in the center of each scatter plot.

Figures S5–S7 **Hexbin plot representing the overlapped sites in the CpG (***n*** = 2715), CHG (***n*** = 111), and CHH (***n*** = 192) regions between male and female pups from the HMFA group in comparison with the LMFA group from all the significant (***P*** < 0.05) differential methylation sites**. Each dot on hexbin plot is one of the overlapped sites. The colors blue, green, yellow and red represent the dot density from lower to higher order, in accordance with the prevalence of the overlapping sites.

Table S1 **Genes in male offspring from high maternal folic acid (HMFA) diet, which exhibited hyper methylation (***P*** < 0.05) compared with genes in male offspring from low maternal folic acid (LMFA) in CpG/CHG/CHH contexts**.

Table S2 **Genes in male offspring from high maternal folic acid (HMFA) diet, which exhibited hypo methylation (***P*** < 0.05) compared with genes in male offspring from low maternal folic acid (LMFA) in CpG/CHG/CHH contexts**.

Table S3 **Genes in male offspring from high maternal folic acid (HMFA) diet, which exhibited hyper methylation (***P*** < 0.05) compared with genes in female offspring from low maternal folic acid (LMFA) in CpG/CHG/CHH contexts**.

Table S4 **Genes in male offspring from high maternal folic acid (HMFA) diet, which exhibited hypo methylation (***P*** < 0.05) compared with genes in female offspring from low maternal folic acid (LMFA) in CpG/CHG/CHH contexts**.

Table S5 **List of primers used in this study**.

## Abbreviations

FA: Folic acid
NTDs: Neural tube defects
LMFA: Low maternal folic acid
HMFA: High maternal folic acid
5 mC: 5-methylcytosine
CB: Cerebellum.
