# Supplementary figures and images for: DNA Methylation Profiling at Single-Base Resolution Reveals Gestational Folic Acid Supplementation Influences the Epigenome of Mouse Offspring Cerebellum
Source: Front Neurosci. 2016 May 3;10:168. doi: 10.3389/fnins.2016.00168 (PMC4854024; doi:10.3389/fnins.2016.00168)

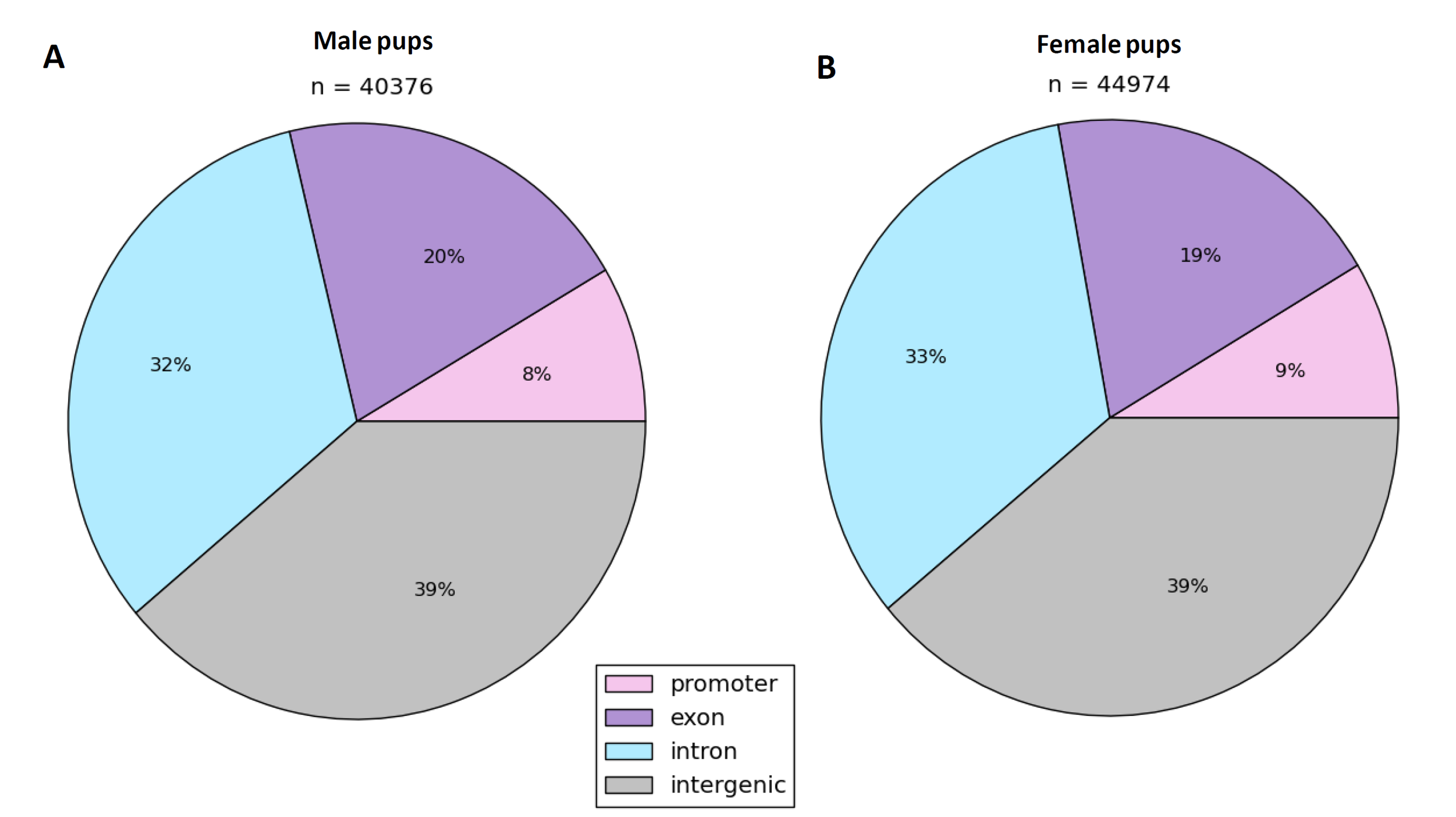

Supplement: Figure S1 — Distribution of differentially methylated sites in CpG island sequences. (A) Male; low maternal folic acid (LMFA) vs. high maternal folic acid (HMFA). (B) Female LMFA vs. HMFA. [file Image1.TIF]

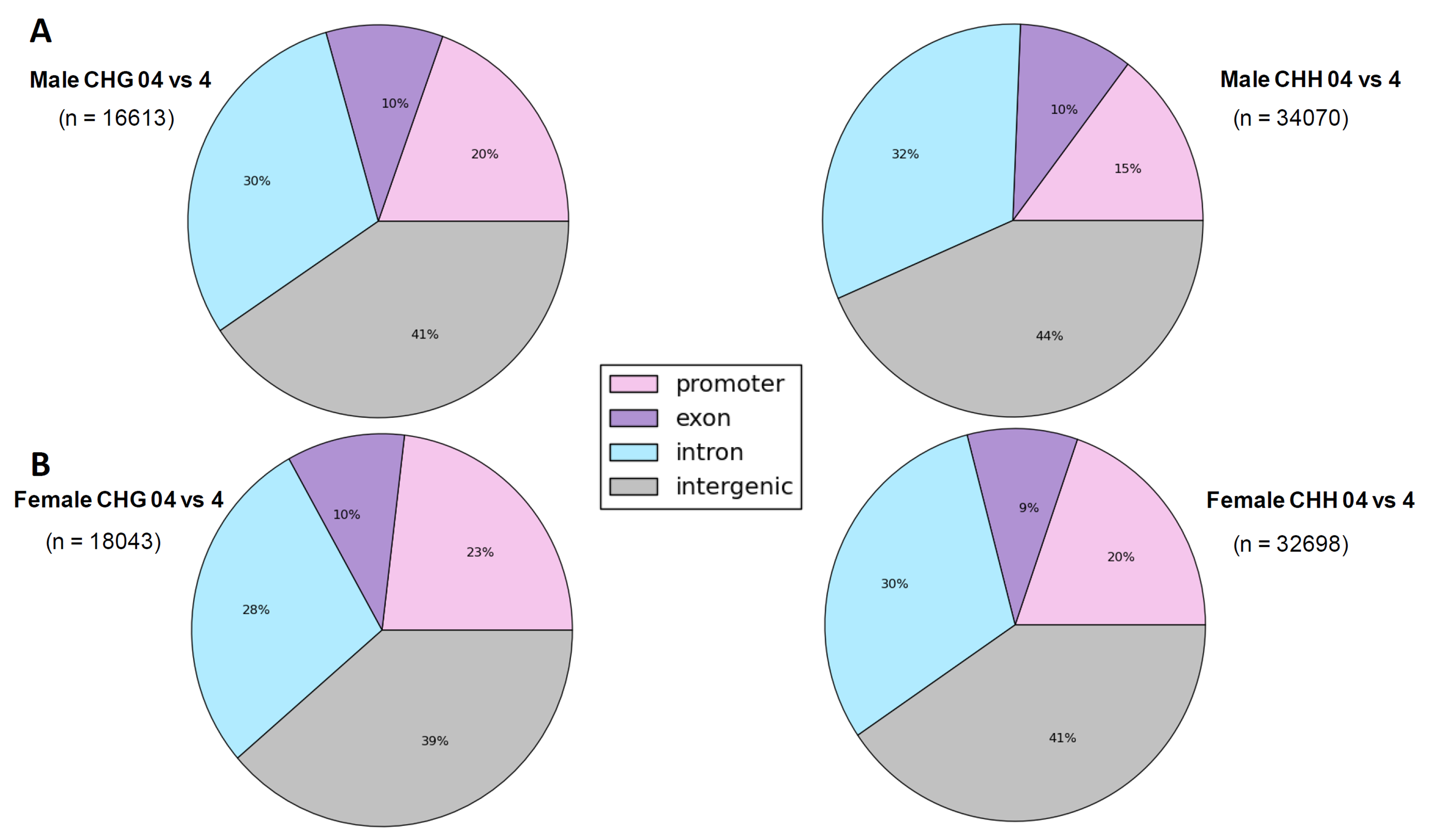

Supplement: Figure S2 — Distribution of differentially methylated sites in non-CpG (CHG/CHH) sites of cerebellum. (A) Male low maternal folic acid (LMFA) vs. high maternal folic acid (HMFA). (B) Female LMFA vs. HMFA. [file Image2.TIF]

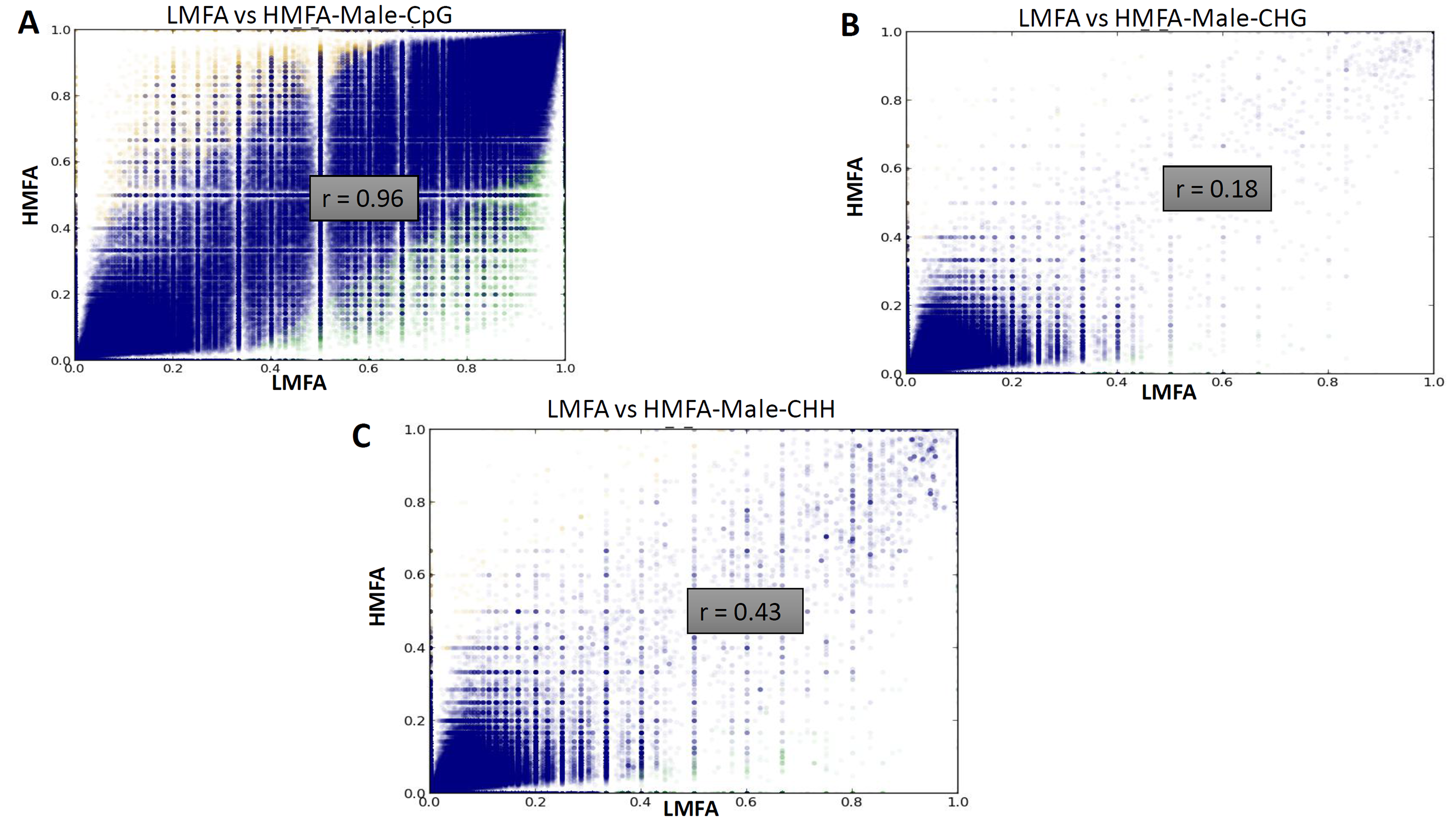

Supplement: Figure S3 — Scatter plot representing the distribution of the methylation ratio for corresponding sites (A) CpG, (B) CHG, and (C) CHH of LMFA vs. HMFA of male pups. Pearson's correlation coefficient is denoted in the center of each scatter plot. [file Image3.TIF]

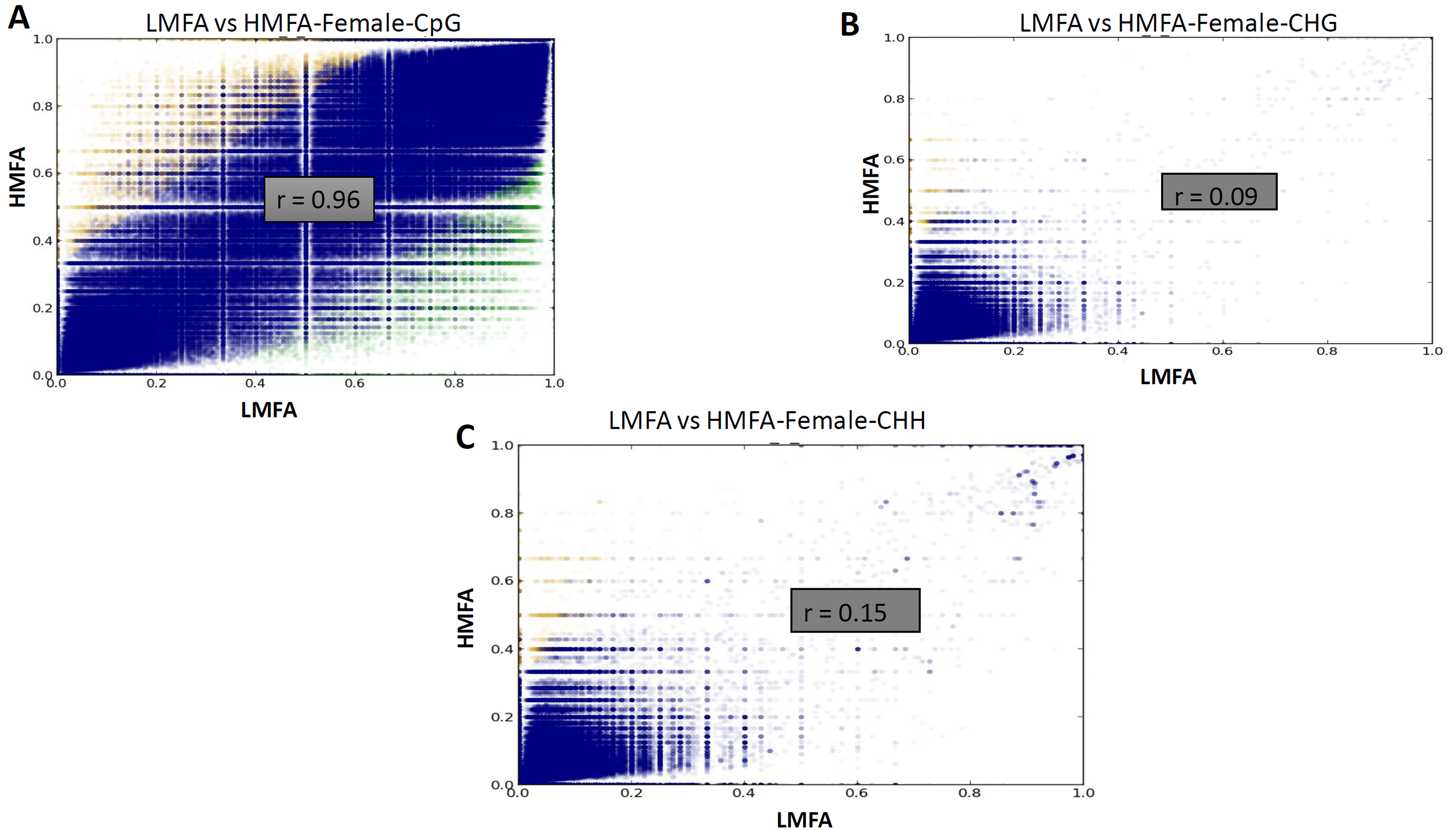

Supplement: Figure S4 — Scatter plot representing the distribution of the methylation ratio for corresponding sites (A) CpG, (B) CHG, and (C) CHH of LMFA vs. HMFA of female pups. Pearson's correlation coefficient is denoted in the center of each scatter plot. [file Image4.TIF]

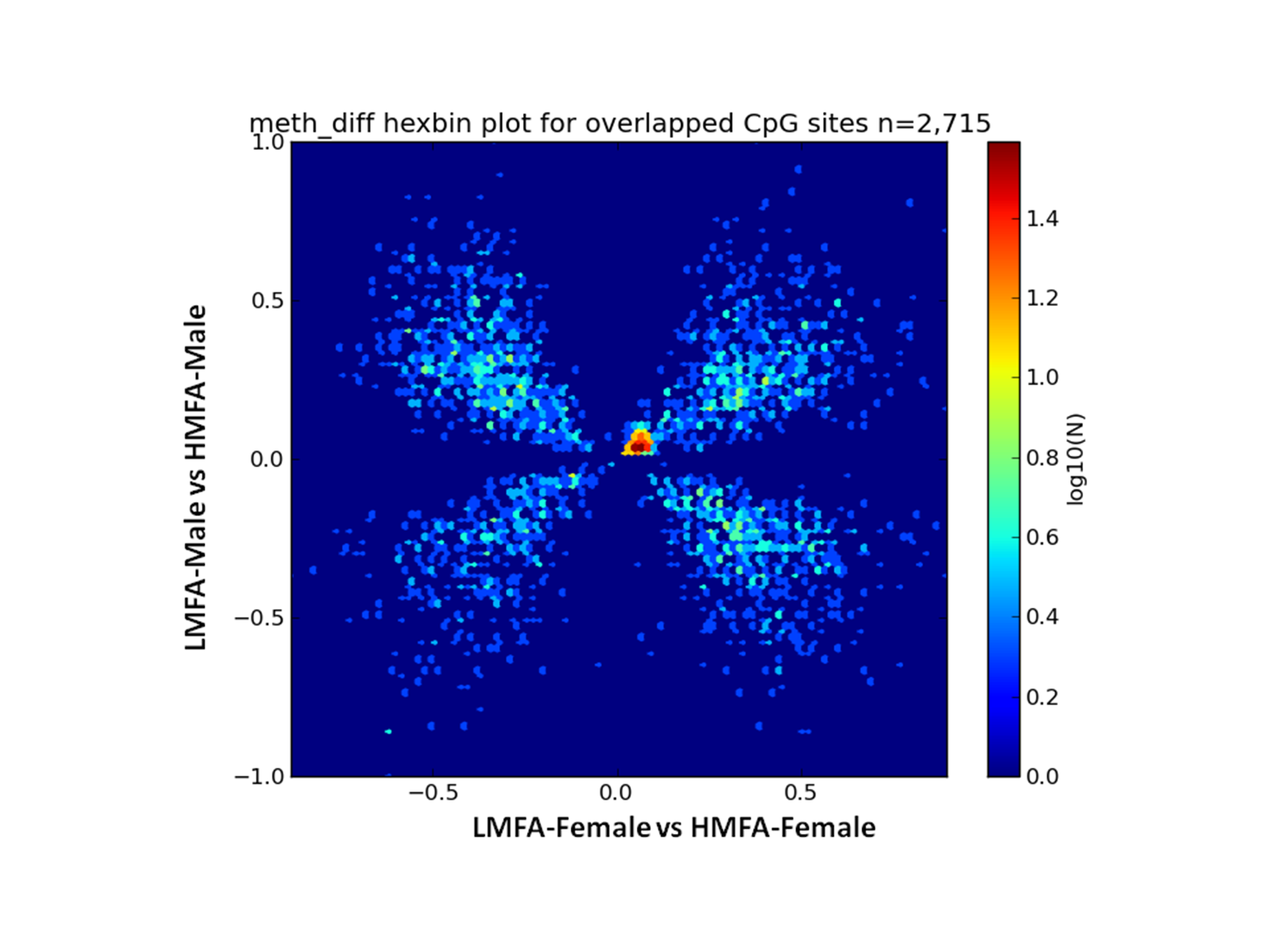

Supplement: Supplementary file 5 [file Image5.TIF]

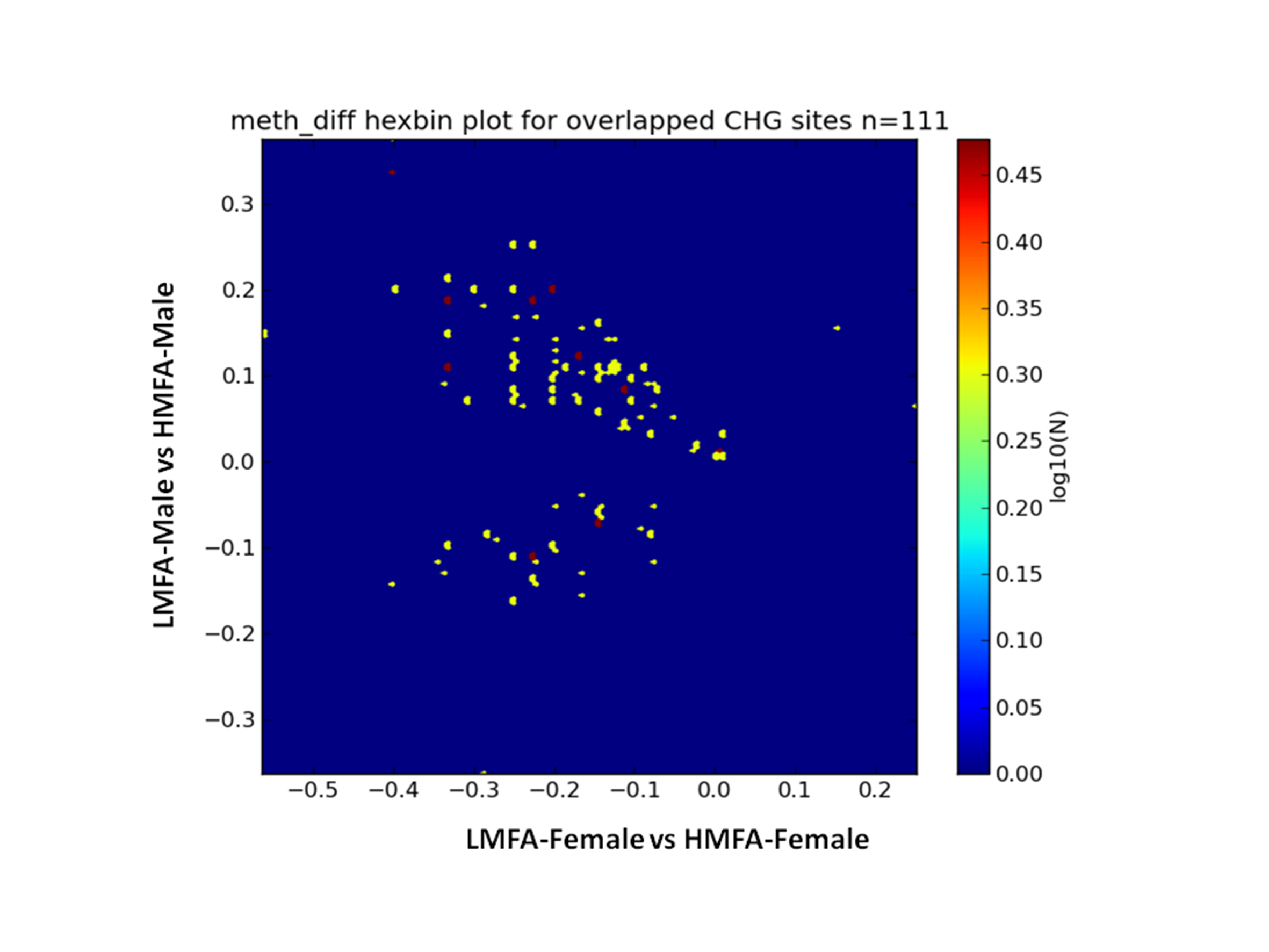

Supplement: Supplementary file 6 [file Image6.TIF]

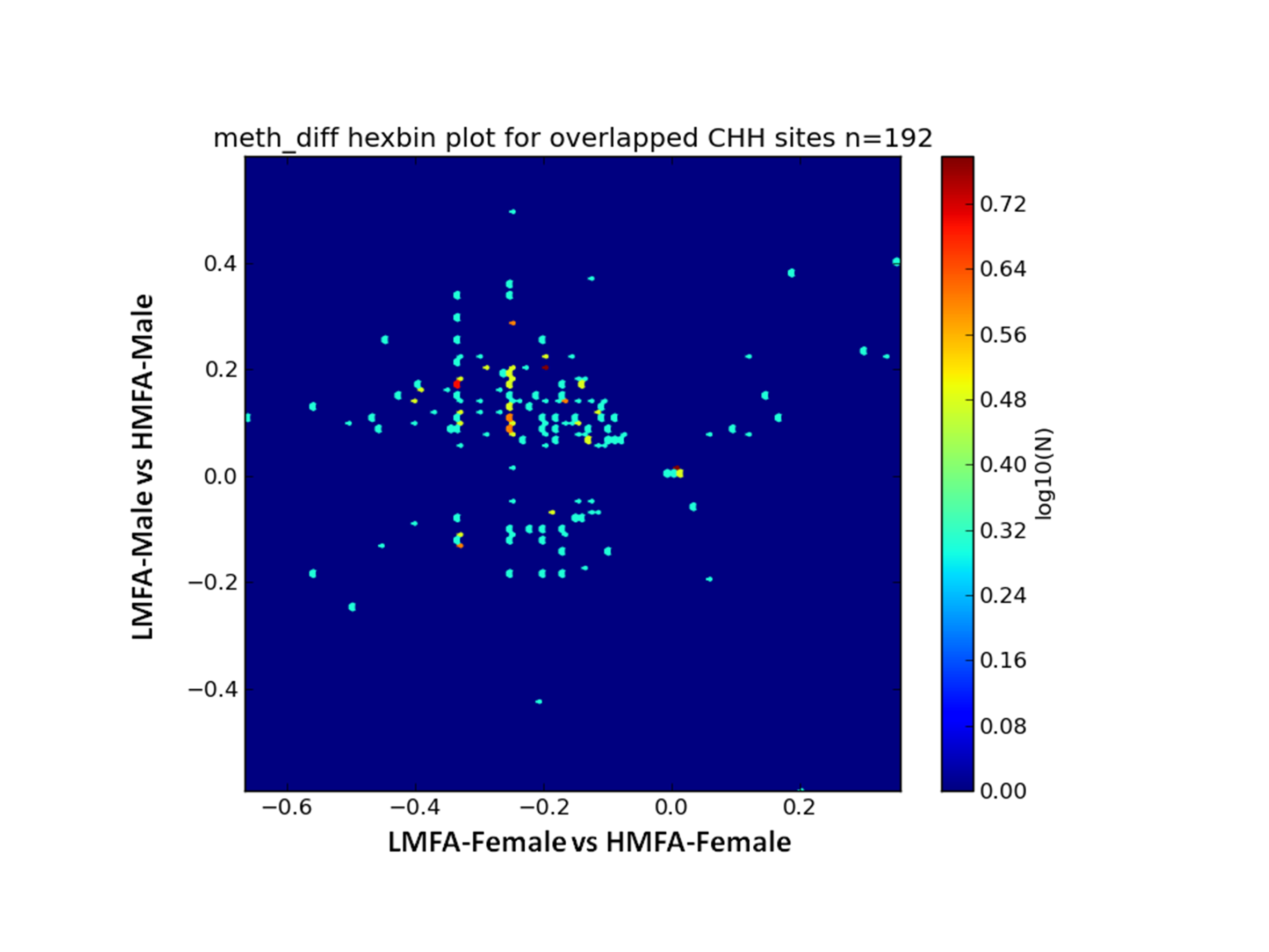

Supplement: Figures S5–S7 — Hexbin plot representing the overlapped sites in the CpG (n = 2715), CHG (n = 111), and CHH (n = 192) regions between male and female pups from the HMFA group in comparison with the LMFA group from all the significant (P < 0.05) differential methylation sites. Each dot on hexbin plot is one of the overlapped sites. The colors blue, green, yellow and red represent the dot density from lower to higher order, in accordance with the prevalence of the overlapping sites. [file Image7.TIF]
