# Supplementary material for: DNA Methylation Profiling at Single-Base Resolution Reveals Gestational Folic Acid Supplementation Influences the Epigenome of Mouse Offspring Cerebellum
Source: Front Neurosci. 2016 May 3;10:168. doi: 10.3389/fnins.2016.00168 (PMC4854024; doi:10.3389/fnins.2016.00168)
Supplement: Table S5 — List of primers used in this study. [file Table5.DOCX]

**Table S5** List of primers used in this study.

| Arid3a-M-F | GCA TGA AGC TGA AAA GGT CA |
| --- | --- |
| Arid3a-M-R | TGG ACC TTT GAG GAG CAG TT |
| Atp1a1-M-F | TAC TTG TCT CGT CCA ACC CC |
| Atp1a1-M-F | TAC TTG TCT CGT CCA ACC CC |
| Atp1a1-M-R | CGG GAG CTG CTC TCT TCT C |
| Atp1a1-M-R | CGG GAG CTG CTC TCT TCT C |
| Bre-M-F | CAA TGA GAC ATC CTC CTC CG |
| Bre-M-R | CCA GGT GTA CCC CAA GTT GT |
| Cacna1a-M-F | TCC GTA GGT CAA ACT CCG TC |
| Cacna1a-M-R | CCA CAA AGG CTC CTA CCT GA |
| Cacna1g-M-F | AGG CTG GTG AAG ACG ATG TT |
| Cacna1g-M-R | TCC TGG TCA ATA CCC TCA GC |
| Celsr3-M-F | GGA GTT GAA AGA GGC AAG GA |
| Celsr3-M-R | CTT CTC AGA GCC AGG GAG G |
| Cntnap2-M-F | TGG GGA AGA CCA GAG ATG AG |
| Cntnap2-M-R | CTG GAT TCT CAG CAG CTT CA |
| Col11a2-M-F | GGG GGT CCC TCT ACA AAC AT |
| Col11a2-M-R | AGT CCC TTG CCA TTC CTT G |
| Coro1a-M-F | ATA GCA CTG GTC AGC CTT GG |
| Coro1a-M-R | GGT CAC GCT CAG CTC TCC |
| Cox10-M-F | TTC ATG TTT GAG TCG AAC GG |
| Cox10-M-R | TTC CTG CTT ACA TCC CTT GG |
| Dcaf17-M-F | ACT TCA CTG CGA TGA CCT CC |
| Dcaf17-M-R | TAT CCG CAA CTA CAG GGG AA |
| Dnmt1-M-F | GCC ATC TCT TTC CAA GTC TTT |
| Dnmt1-M-R | TGT TCT GTC GTC TGC AAC CT |
| Dnmt3a-M-F | GCT TTC TTC TCA GCC TCC CT |
| Dnmt3a-M-R | CCA TGC CAA GAC TCA CCT TC |
| Dnmt3b-M-F | CTG GCA CCC TCT TCT TCA TT |
| Dnmt3b-M-R | ATC CAT AGT GCC TTG GGA CC |
| Drp2-M-F | AGA CCA ACT GTG GAG CAT CA |
| Drp2-M-R | ATT CAC AGA GGC AGC CTA GC |
| Fmr1-F | TCTGTCTCTCTGGTTGCCAGT |
| Fmr1-R | CCAATGGCGCTTTCTACAAG |
| Gabrb1-M-F | AAG CCC CAA ACT CTC TCG AT |
| Gabrb1-M-R | GCC GAC TAA GTT GCA TTC CT |
| Gabrg3-M-F | GTT GAA TCG AAG GCG ACT GT |
| Gabrg3-M-R | ACA GCA TTG GTC CTG TGT CA |
| Gabrr1-M-F | GAG CTT CGT CTC AGG ATT GG |
| Gabrr1-M-R | GAG CCA TCT AGG AAA GGC AG |
| Gad1-M-F | CCA TCC AAC GAT CTC TCT CA |
| Gad1-M-R | GCT GAC ATC GAC TGC CAA TA |
| Gfra1-M-F | TTC TTG CAG GTG TCA TCC AG |
| Gfra1-M-R | GGC AGT CCC GTT CAT ATC AG |
| Grik4-M-F | TGG GGT CAT CCA AGA TAG CA |
| Grik4-M-R | TAG AAG ATG CCC CGT GTC TC |
| Grin2b-M-F | GCC AAA CTG GAA GAA CAT GG |
| Grin2b-M-R | TCT GCT CAG ACT CTC ACC CC |
| Grip1-M-F | ATA CCG TCA GGC CAA GAG TG |
| Grip1-M-R | TCT GCT TAC AAG CGG AGG AG |
| Gtf2i-M-F | CGT GCT CTT TAG CCT CTT GG |
| Gtf2i-M-R | TGT CCC TTC AGA AAC CAG TG |
| Itga2-M-F | AAC TGT TCA CTT GAA GGC CC |
| Itga2-M-R | GCG GCT GCT AAT GCT AGT TC |
| Kcnab2-M-F | GAC AGA TCA GCG TGC CTT TT |
| Kcnab2-M-R | AGG GCA GGA AGG AAG ATC C |
| Kcnb1-M-F | TGT TGA GTG ACA GGG CAA TG |
| Kcnb1-M-R | CTG GAG AAG CCC AAC TCA TC |
| Kcnk10-M-F | AGA GTC GAG GAA CAG GGT GA |
| Kcnk10-M-R | AGA AAG CAG GTG AAC TGG GA |
| Kcnq1-M-F | TAC AAA GTA CTG CAT GCG CC |
| Kcnq1-M-R | GAC CTG GAA GGG GAG ACA CT |
| Kcnq2-M-F | AGA AAG CAC AAG GCA GGA GA |
| Kcnq2-M-R | GCA AGC TGC AGA ATT TCC TC |
| Kcnq4-M-F | GGA AAC CCT TCT GTG TCA TCG A |
| Kcnq4-M-R | TGT GCC CGC AGC TAT CAC T |
| Myst4-M-F | ACA TAT TGG AAT GGG ATC GG |
| Myst4-M-R | CCA GTA CCC CAG TGC TTT TC |
| Nrxn2-M-F | ACT TCG CTG TTT AAC GTC AGG |
| Nrxn2-M-R | ACT GCA GCC ACA CTG GAT TT |
| Ntrk2-M-F | CCC AGC CTT TGT CTT TCC TT |
| Ntrk2-M-R | AGT TGG CGA GAC ATT CCA AG |
| Park2-M-F | ATC GAC CTC CAC TGG GAA G |
| Park2-M-R | GCG TAG GTC CTT CTC GAC C |
| Pfn1-M-F | GGT CTT TGC CTA CCA GGA CA |
| Pfn1-M-R | CGT AGG CTA CAA GGA CTC GC |
| Plxna4-M-F | AGG CCA GTG AGT GGT TCT TG |
| Plxna4-M-R | CTC TGG GAG TTT CCG AAA TG |
| Rbfox1-M-F | TAA GGC TGA GCC ATT GTG TC |
| Rbfox1-M-R | GGT TTT GAG GCA ACA GAT GAA |
| Runx1-F | GGA TCC CAG GTA CTG GTA GGA |
| Runx1-R | GCC ATC AAA ATC ACA GTG GA |
| Rxfp2-M-F | GAA AAT ACC CTT TGG GGC AC |
| Rxfp2-M-R | CGC AAT GTG GCT CCT ACT TC |
| Scn8a-MF | ATG TAC AAG GCA GGA GTG GC |
| Scn8a-MR | CTG GAG GAC TTT GAC CCG TA |
| Sgk1-M-F | CCG TGT TCC GGC TAT AAA AC |
| Sgk1-M-R | GTC CTC CAT AAG CAG CCG TA |
| Slc25a12-M-F | GAG TCA TGT AAT GCT CCC CG |
| Slc25a12-M-R | GGT CAA GGT GCA TAC AAC CA |
| Unc5b-M-F | CCA ATA GGA AGT ACG GCA GC |
| Unc5b-M-R | CGA CAC CGA GCC TAG CAG |
| Hprt1; Dock4; Mid1 | Source SA Biosciences (Frederick, MD, currently a Qiagen Co). |
